# New potentials in biomedical application of hydrated electrons: A functional and biomedical effects study of an electromagnetic base liquid containing hydrated electrons

**DOI:** 10.1371/journal.pone.0336479

**Published:** 2025-11-24

**Authors:** Zhuangbin Zheng, Fanlei Ran, Lushan Liang, Yiqian Zhang, Xianwen Li, Liqun Zhang, Lijun Bi

**Affiliations:** 1 State Key Laboratory of Respiratory Disease, The First Affiliated Hospital of Guangzhou Medical University, Guangzhou Medical University, Guangzhou, China; 2 Guangzhou National Laboratory, Guangzhou, China; 3 Beijing Chest Hospital, Capital Medical University, Beijing, China; 4 Institute of Biophysics, Chinese Academy of Sciences, Beijing, China; 5 Lead contact; SRM University AP SEAS: SRM University AP School of Engineering and Sciences, INDIA

## Abstract

Hydrated electrons (e_aq_^-^) are widely studied in pollutant degradation owing to their high reducing power. Recent studies indicate that transiently generated e_aq_^-^ during radiotherapy can enhance chemotherapeutic antitumor effects via reduction activity. However, biomedical applications remain limited because conventional methods generate e_aq_^-^ in situ and are short-lived, precluding storage. In this study, we present a storable electromagnetic base liquid (EBL), reference-linked to prior preparation work, and analyze its physicochemical and organism-level effects. The EBL showed strong alkalinity (pH 13.08), low oxidation-reduction potential (ORP 47.1 mV), and a total antioxidant capacity of 1.6 mM Trolox equivalents antioxidant capacity. Using a larva zebrafish (*Danio rerio*) digital phenotyping platform, we identified a non-adverse concentration and generated unbiased predictions clustering EBL with antitumor, uric acid-lowering, and hypoglycemic drugs. These findings motivate further biological investigation of EBL’s biomedical potential and provide a basis for subsequent validation.

## Introduction

As reducing agents play a pivotal role in scavenging reactive oxygen species (ROS) and maintaining redox homeostasis in biological systems, the identification of novel reductants is now vital for developing more efficacious therapeutic strategies [1–3]. The hydrated electron (e_aq_^-^), an electron solvated within a water cage, is one of the most potent reductants [4,5]. Progressing from studies in ammonia solutions to flash photolysis in water, early work established the existence and spectrum of the e_aq_^-^ (e.g., a broad band near 720 nm) [6–9]. Since the confirmation of the existence of the e_aq_^-^, given its exceedingly high reducibility, as characterized by a standard reduction potential of approximately −2.9 V, it has been widely researched and applied in environmental remediation [10].

The e_aq_^-^ can effectively degrade or transform a variety of refractory pollutants through reduction reactions. Their applications include the reduction of inorganic substances (such as nitrate, perchlorate, and bromate), the degradation of organic compounds (including ketones, phenols, alkenes, and amino acids), and more recently, the remediation of structurally complex and environmentally persistent emerging contaminants [11–17]. Beyond remediation, biomedical interest is growing—for example, radiation-generated e_aq_^-^ can activate N-oxide prodrugs in tumors [18]. However, a major limitation is that e_aq_^-^ can only be generated in situ and is highly transient, hindering its biological study and practical use [19–22].

In this context, we prepared an electromagnetic base liquid (EBL), an aqueous containing e_aq_^-^ system whose preparation method and reducing characteristics are documented in a granted patent [23]. In the current study, we examined its biological relevance using a combination of analytical techniques and a larval zebrafish model. As a vertebrate discovery model, zebrafish offers organism-level, multi-system readouts with strong translational relevance, and data-driven clustering can position unknown samples relative to reference compounds with known mechanisms of action [24,25]. Specifically, we (i) quantified key physicochemical properties—including pH, oxidation-reduction potential (ORP), Trolox-equivalent antioxidant capacity (TEAC), and total dissolved solids (TDS)—and (ii) conducted an organism-level, hypothesis-generating screen in larval zebrafish by integrating high-content phenotyping with machine-learning-based functional clustering. By introducing a storable, reducing aqueous system into a biological characterization setting, this study offers quantitative descriptors and unbiased phenotypic signatures, laying the groundwork for further mechanistic and mammalian validation.

## Materials and methods

### Materials and apparatus

Total antioxidant capacity (TAOC) kit and crystal violet staining solution were purchased from Beyotime Biotechnology Co., Ltd. The pH of EBL was measured using a pH/ATC electrode (PY-P50, Sartorius, GER), and the ORP was measured using the ORP machine (9179BNMD, ThermoFisher Scientific, USA). TDS were detected using a conductivity meter (DDBJ‑351L, Shanghai Leici, China). The absorbance was measured on the BioTek Epoch microplate spectrophotometer (Agilent Technologies Inc.).

### EBL preparation

The detailed method for preparing EBL has been described in the patent [23]. 400 g of silicon powder and 400 g of NaOH particles were added to an open reactor along with one part of ammonia water (20 g ammonia and 400 g water) and stirred for one hour. When the preliminary reaction mixture started bubbling and the temperature reached 110 °C, a second part of ammonia water (20 g ammonia and 200 g water) was added. The reaction temperature was controlled not to exceed 160 °C for 4 h. 20 g of distilled water was added to the complete reaction mixture, and the diluted mixture was filtered to remove impurities. After letting the mixture stand for 10 h, a negative polarity voltage was applied to the tungsten steel electrode of a plasma discharge reactor using a power supply (DC, 20 kV, 50 mA), and a discharge plasma was generated between the electrode and the liquid surface. A 2 mA discharge current was applied for 10 min to obtain the gray-blue EBL.

### pH and ORP measurements

ORP is a measure of the oxidative or reductive capability of a solution. It reflects the macroscopic oxidative or reductive nature of all substances in solution, indicating a tendency for electron transfer [26,27]. We measured the pH and ORP values of EBL at different concentrations. The EBL was diluted with distilled water to prepare 2-mL solutions at 0.001%, 0.01%, 0.1%, 1%, 10%, and 100% (v/v). The pH meter was routinely calibrated with standard buffers (pH 4.01, 6.86, and 9.18), and the ORP meter was calibrated using the ORP standard solution. To determine the effect of temperature on the EBL, the solution was placed in a 50 °C incubator for 24 h and subsequently allowed to cool to room temperature. Next, the pH and ORP of 0.01%, 1%, and 100% EBL were measured. To test the temporal stability of EBL at room temperature, the pH and ORP of 0.01%, 1%, and 100% EBL were measured after storing for 0, 2, 4, and 8 weeks. Different storage conditions and times were used to detect unused EBL. Distilled (0% EBL) water was used as a control, with three parallel replicates for each concentration tested.

### Determination of TAOC

The TAOC was determined using the 2,2’-azino-bis (3-ethylbenzothiazoline-6-sulfonic acid) (ABTS) radical cation decolorization assay, as described by Re et al. (1999) with minor modifications [28]. According to the manufacturer’s instructions, the working solution was prepared by mixing ABTS and potassium persulphate solutions in a 1:1 ratio. The mixture was stored for 18 h at room temperature before use. The absorbance of the working solution at 734 nm was adjusted to 0.7 ± 0.05 by adding 1 × phosphate-buffered saline (PBS) solution. Then, 10 μL of distilled water or EBL (0.5%, 1%, 1.5%, 2%, 2.5%, 3%, 40%) was mixed with 200 μL working solution and added to the 96-well plate. After 6 min of incubation, the absorbance was measured at 734 nm using the microplate spectrophotometer. Trolox was used as the reference compound, and the results were expressed as mmol of TEAC per liter of the solution.

### TDS determination

TDS is defined as the total mass concentration of dissolved inorganic ions and organic matter in an aqueous solution [29]. The TDS values were detected using the conductivity meter in the TDS mode. Saturated potassium chloride solution was used to calibrate the electrode. TDS readings were recorded for EBL diluted to 0.5%, 1%, 2%, 3%, 4%, 8%, 10%, and 20%. Each concentration was measured in triplicate.

### Safety evaluation

According to the EU Directive 2010/63/EU, the zebrafish (*Danio rerio*) larvae less than 5 days post fertilization (dpf), before the independent feeding stage, can be considered as *in vitro* models [30–32]. Wild-type zebrafish larvae with normal development at 4 dpf were randomly distributed into 6-well plates (15 larvae per well; three replicate wells). 0.011%, 0.022%, 0.044%, 0.088%, 0.17%, 0.35%, and 0.5% EBL were added into the culture system of zebrafish larvae and incubated at 28 °C in a biochemical incubator for 4 days. The survival and malformation of zebrafish larvae were observed daily, and the maximum safe concentration in the experimental group was obtained for further experimentation.

### Function prediction

The potential effects of EBL on zebrafish larvae were determined as previously described. Randomly selected normal 3 dpf zebrafish larvae (Elavl3:H2B-GCaMP6f) were incubated in 6-well cell culture plates with EBL and served as the test group. The control group had no EBL added. Three days later, the plates were imaged on an automated inverted fluorescence microscope (NIB950-FL). T‐score brain activity map (BAM), tail flicking, and heart rate were analyzed as previously described. The difference in calcium transient counts between the control and test groups was calculated and projected (by summing along the Z-axis) onto a two-dimensional surface to construct a BAM, reflecting brain activity in zebrafish larvae. BAMs were obtained from five individual zebrafish larvae for each test article, and the comprehensive T-score for BAM was calculated [33,34]. Cluster analysis of the brain nerve T-score BAM, behavioral data, cardiac beat data, and drug comprehensive database was conducted using a machine learning algorithm.

### Statistical analysis

Data were statistically analyzed using GraphPad Prism 8. All experiments were repeated thrice, and the data are presented as mean ± standard deviation (SD). Safety evaluation data were analyzed using the chi-square test. Behavioral and cardiac response data were analyzed using two-tailed unpaired t-tests. All other datasets were analyzed using one-way ANOVA followed by appropriate post hoc tests. A *P* value < 0.05 was considered statistically significant.

## Results

### EBL exhibits high pH, low ORP, and long-term storage stability

**Fig 1:**
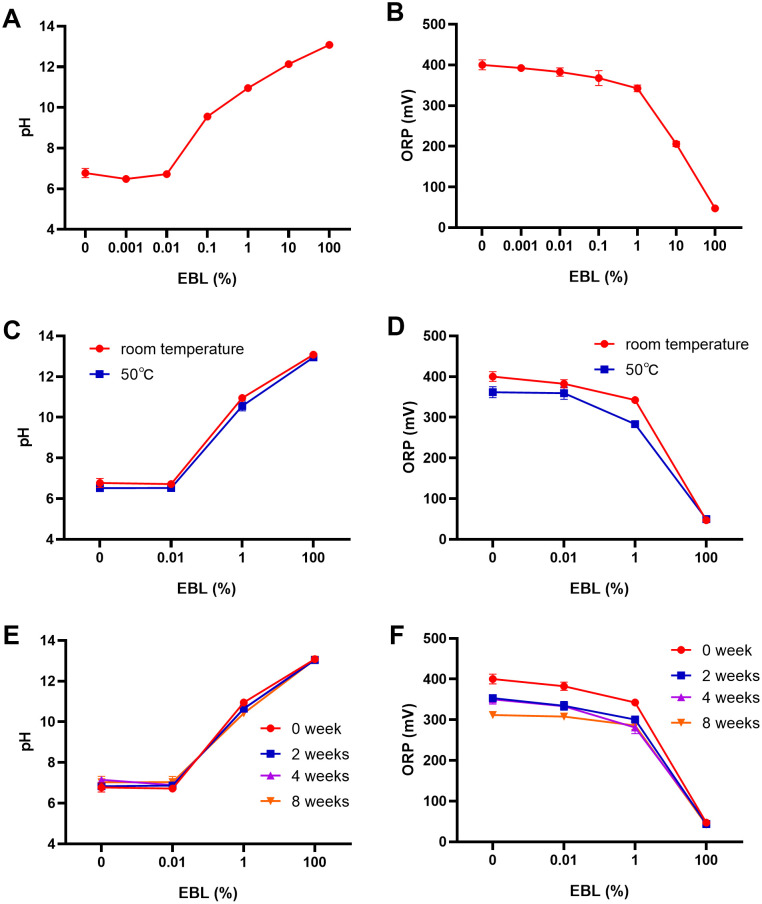
pH and ORP profiles and stability of EBL. **(A)** pH across EBL concentrations, **(B)** ORP across concentrations, **(C)** pH after 24 h at 50 °C, **(D)** ORP after 24 h at 50 °C; **(E)** pH after storage at room temperature for 0, 2, 4, and 8 weeks, **(F)** ORP after storage at room temperature for 0, 2, 4, and 8 weeks. Unless indicated otherwise, data are mean ± SD from n = 3 independent replicates per condition. EBL concentrations are % (v/v).

To profile the physicochemical properties of EBL, we measured pH and ORP across a concentration series. As shown in Fig 1A, pH was highly concentration-dependent. Undiluted EBL had pH 13.08 and decreased upon dilution, most steeply between 0.01% and 0.1%. By contrast, ORP increased with dilution (Fig 1B). Specifically, at 100% EBL, ORP was low 47.1 mV versus 400.1 mV for distilled water. ORP rose steadily with dilution, with the sharpest change between 0.1% and 1% (Fig 1B).

We next assessed pH/ORP stability after heat exposure and room-temperature storage. After 24 h at 50 °C, pH was largely unchanged across concentrations (Fig 1C). Under the same conditions, ORP decreased slightly in diluted samples and in water, whereas undiluted EBL remained < 50 mV (Fig 1D). After 0, 2, 4–8 weeks at room temperature, pH changed minimally (Fig 1E), and ORP also remained relatively stable, with less fluctuation observed at higher EBL levels (Fig 1F). Overall, the dilution series showed small ORP drifts after heat or storage, while pH was essentially stable; undiluted EBL maintained both pH and ORP, consistent with greater storage stability.

### EBL shows a dose-dependent ABTS· ⁺ scavenging activity with a high TEAC value

**Fig 2:**
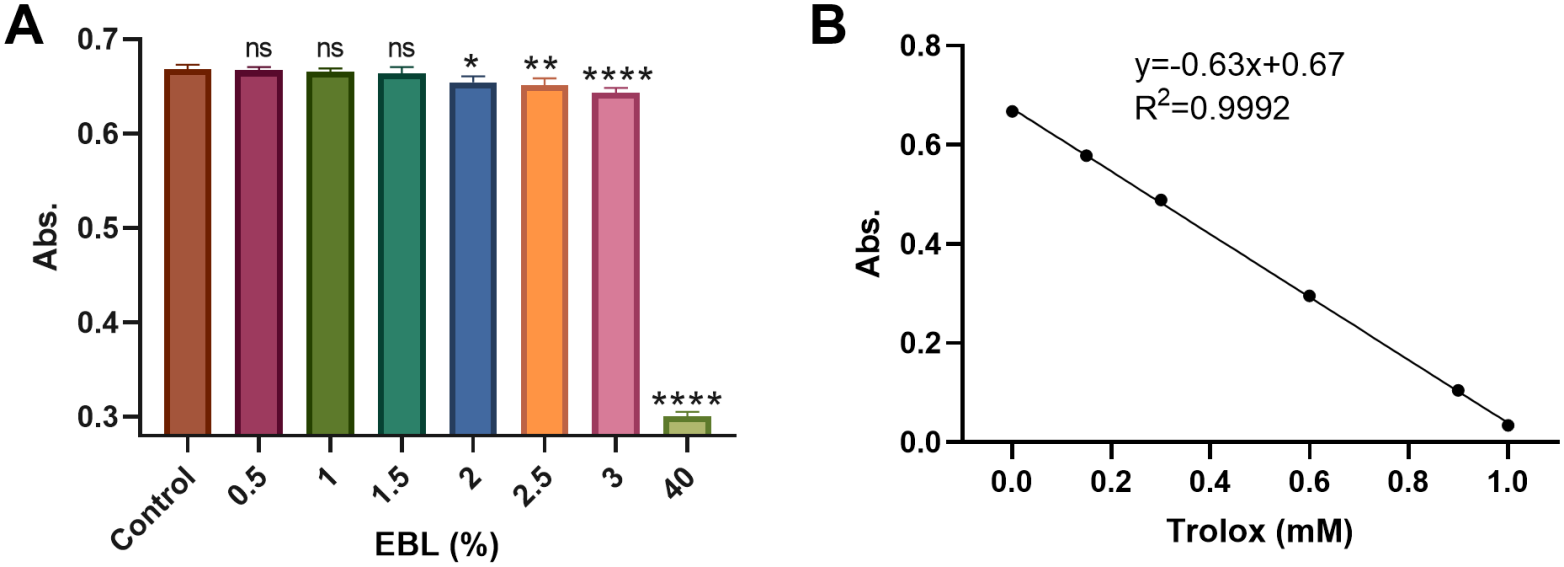
ABTS assay of EBL. **(A)** ABTS• ⁺ decolorization (734 nm absorbance) across indicated EBL concentrations, **(B)** Trolox calibration used to derive TEAC (Abs = −0.63 × c[Trolox] + 0.67, R² = 0.9992). Data are mean ± SD; n ≥ 3 per concentration unless noted. Group comparisons used one-way ANOVA with post-hoc testing; *P* < 0.05 versus water control was considered statistically significant. Significance: *P* < 0.05 (*), *P* < 0.01 (**), *P* < 0.0001 (****), ns = not significant. TEAC is reported as a quantitative descriptor of total antioxidant capacity.

The ABTS· ⁺ decolorization was measured across an EBL concentration gradient. As shown in Fig 2A, EBL produced a concentration-dependent decrease in absorbance, and values differed from water control at ≥2% (*P* < 0.05). To quantify this activity, a Trolox standard curve was established (Abs = −0.63 × c[Trolox] + 0.67; R² = 0.9992; Fig 2B). Based on the calibration, undiluted EBL yielded 1.6 mM TEAC. This value is significantly higher than that of the negative control and exceeds the established TEAC value of 1 mM ascorbic acid (0.99) [35].

### EBL shows high TDS increasing with concentration

**Fig 3:**
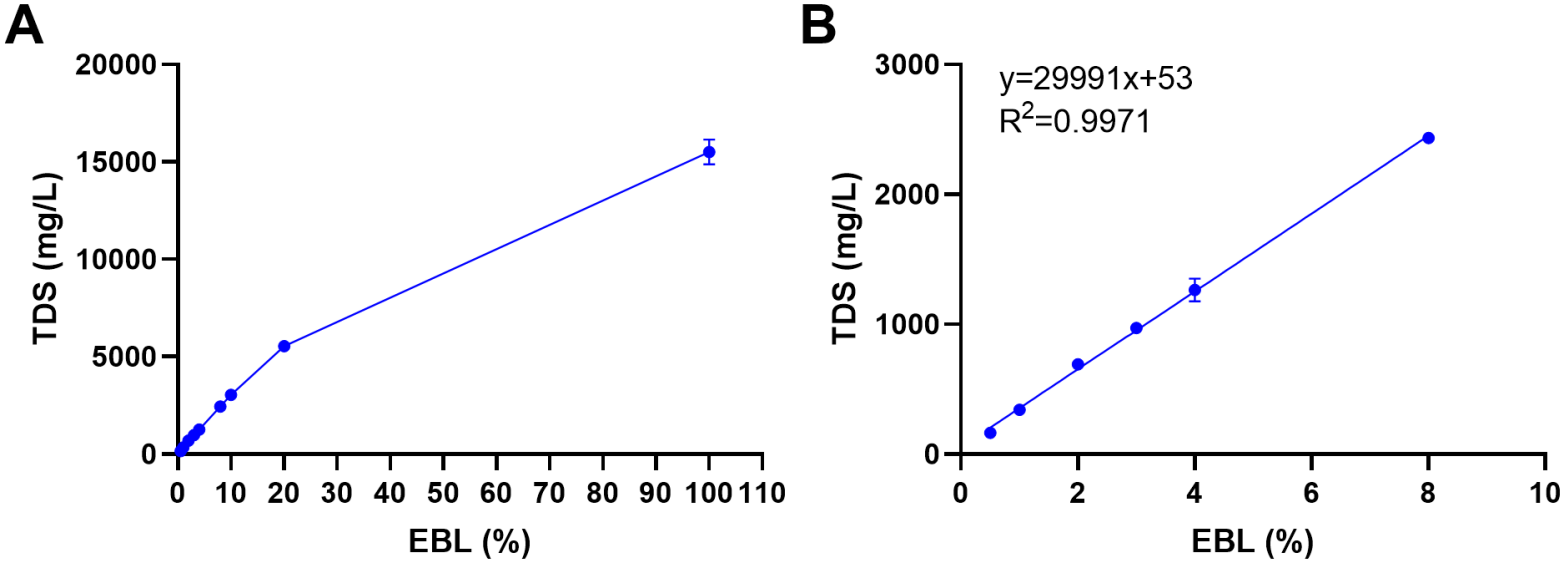
TDS as a function of EBL concentration. **(A)** TDS values across EBL concentrations, **(B)** Linear relation between TDS and concentration at 0.5% − 8% (y = 29,991x + 53; R^2 ^= 0.9971). Data are mean ± SD, n = 3 per concentration (conductivity meter in TDS mode). EBL concentrations are % (v/v).

To further characterize the solution, its TDS were quantified. TDS increased with concentration, reaching 15,513 mg/L at undiluted EBL (Fig 3A). Between 0.5% to 8%, TDS scaled linearly with concentration (R² = 0.9971) (Fig 3B). Extrapolating the low-range linear fit (y = 29,991x + 53) suggests that ≈3.2% EBL would correspond to TDS ≈ 1,000 mg/L, a commonly used benchmark in drinking-water guidelines.

### Integrated zebrafish phenotyping and machine-learning workflow predicts EBL therapeutics

**Fig 4:**
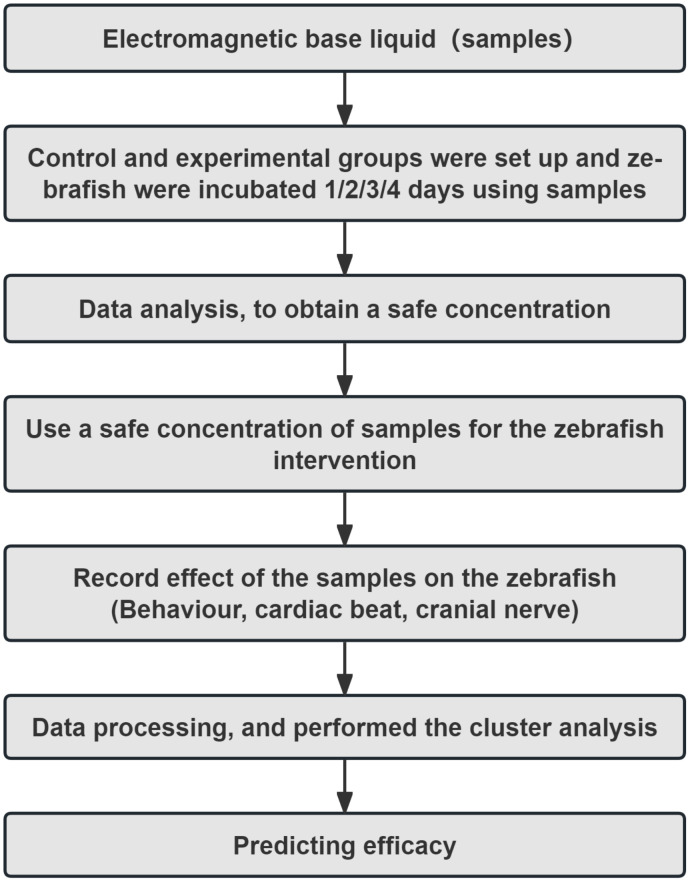
Integrated workflow for organism-level phenotyping and machine-learning inference. Workflow schematic: (i) establish a non-adverse exposure using hatching, malformation, and survival endpoints; (ii) record behavioral (tail-swing), cardiac (heart rate), and brain-wide neural activity readouts; (iii) integrate features and perform machine-learning-based clustering against a reference drug database to infer functional similarity.

To systematically evaluate the efficacy and biological effects of EBL, we implemented an integrated workflow that first established a non-adverse exposure, then measured behavioral, cardiac, and brain-activity readouts, and finally applied machine-learning clustering for functional inference (Fig 4). Specifically, the workflow began with safety assessment using hatching, malformation, and survival assays to establish a safe concentration. We then examined behavioral, cardiac, and neural responses at the safe dosage. Finally, these multidimensional data were integrated via machine learning to cluster EBL with clinically established drugs and predict its potential therapeutic functions.

### Zebrafish larvae safety assessment identifies a non‑adverse exposure under the tested conditions

**Fig 5:**
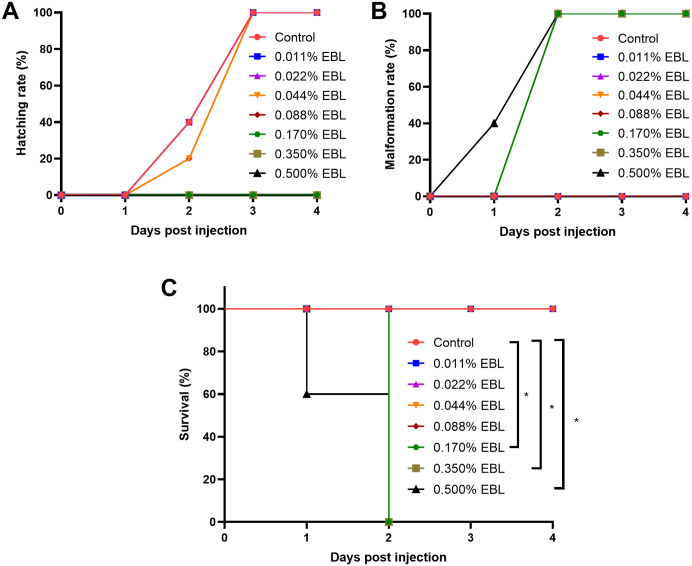
Safety assessment of EBL in zebrafish larvae identifies a non-adverse exposure. **(A)** Hatching rate, **(B)** Malformation rate, **(C)** survival over 4 days at 28 °C across EBL concentrations. Data are mean ± SD at each time point. Significance was assessed by chi-square test (two-sided); *P* < 0.05 (*) considered significant. Under these conditions, 0.088% EBL represents the MNAC.

The effects of EBL concentration on the hatching, malformation, and survival of zebrafish larvae were assessed to determine a safety threshold. The hatching rate reached 100% after 3 and 4 days of incubation at 0.088% EBL (Fig 5A). The malformation rate was 0% at 0.088% EBL but increased to 100% at higher concentrations (Fig 5B). After 2 days of exposure to 0.170%, 0.350%, and 0.500% EBL, all zebrafish larvae died, whereas those treated with 0.088% EBL survived (Fig 5C). These endpoints identified 0.088% as the maximal non-adverse concentration (MNAC) under the present assay conditions.

### EBL clusters with antitumor, uric acid-lowering, and hypoglycemic drugs via machine learning analysis

**Fig 6:**
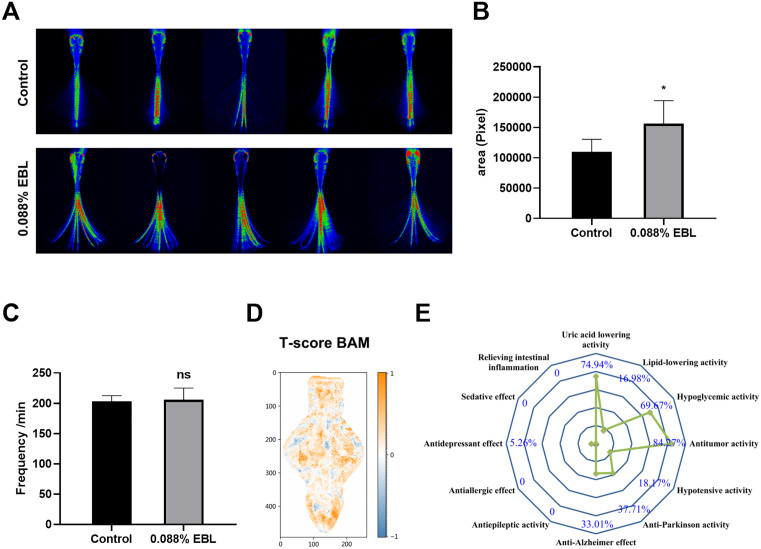
Behavioral, cardiac, and brain-activity responses to EBL with machine-learning-based functional clustering. **(A)** Representative depiction of zebrafish larvae tail movement, **(B)** Tail-swing area metric (area per movement frequency); **(C)** Heart rate (beats per minute); **(D)** T-score BAM at 0.088% EBL (color scale encodes T-score magnitude, with orange representing increased and blue indicating lower activity), **(E)** Radar plot of similarity between the EBL phenotype and reference drugs classes derived from standardized feature vectors. EBL (green line) shows high similarity to antitumor, uric acid-lowering, and hypoglycemic drugs. Concentric circles indicate similarity from low (center) to high (periphery). Data are mean ± SD. Two-tailed unpaired t-tests were used for behavior and heart rate; *P* < 0.05(*) was considered significant (ns, not significant). Similarity scores in panel E reflect clustering outputs and do not denote statistical significance.

To assess the potential biological effects of EBL, we examined its impacts on behavior, cardiac function, and cranial neural activity in zebrafish larvae within a safe concentration range. These phenotypic data were subsequently used for functional prediction of EBL using machine learning. At the MNAC (0.088%), EBL increased the tail-swing frequency (*P* < 0.05) without altering heart rate (Fig 6A-6C). BAM analyses indicated brain-wide activity changes, with higher T-scores across forebrain, midbrain, and hindbrain regions (Fig 6D). Based on these phenotypic data, a machine learning-based cluster analysis was performed against an integrated drug database. Feature-based clustering mapped EBL near reference profiles for antitumor, uric acid-lowering, and hypoglycemic drugs (similarity 84.27%, 74.94% and 69.67%, respectively; Fig 6E).

## Discussion

The chemistry mediated by e_aq_^-^ has long enabled powerful reductive processes in aqueous systems, especially for the degradation of halogenated compounds, nitrates, and emerging contaminants [36–39]. More recently, interest has grown in leveraging these redox properties for biomedical purposes, including site‑specific prodrug activation and protein redox modulation [18,40,41]. Within this evolving context, we introduce a storable, redox‑active alkaline solution prepared via a patented method [23] and evaluate its biological effects through physicochemical profiling and vertebrate‑model screening. Taken together, the findings suggest that such a matrix can serve as a tractable platform for redox‑based biomedical hypothesis generation.

At the physicochemical level, the solution exhibited a low ORP and a quantifiable TEAC, consistent with bulk reducing capacity. ORP is widely used in clinical and environmental settings as an aggregate index of overall redox balance [26,42,43], and ABTS• ⁺ decolorization is an established approach to approximate antioxidant capacity in aqueous matrices [28,44]. We interpret these as complementary system‑level indicators rather than proof of specific molecular carriers. The combination of stable redox properties and compatibility at sub‑percent dilutions in organism‑level assays supports its utility for in vivo investigation.

At the organism level, we leveraged larval zebrafish for multisystem screening, including behavioral, cardiac, and brain‑activity readouts. This model has demonstrated predictive value in toxicology and early pharmacology and supports high‑content phenotyping coupled with machine‑learning‑based inference [24,33,34,45–51]. The induced phenotypic profile mapped near drug spaces relevant to cancer, uric‑acid regulation, and glycemic control, aligning with the recognized roles of oxidative stress and redox imbalance in these disease areas [52–54]. Redox nodes such as H₂O₂ signaling and the GSH/GSSG couple are known to shape cellular decisions across these contexts, providing a coherent rationale for testing redox‑active matrices in disease‑relevant models [52,53]. In parallel, pharmacological strategies that co‑modulate redox tone alongside primary therapeutic mechanisms strengthen the translational logic for such testing [55,56]. Consistent with this rationale, preliminary unpublished work from our group indicates that, in a murine model of hyperuricemia, EBL reduced serum uric acid and free‑radical readouts while increasing glutathione levels, findings consistent with urate‑lowering and antioxidant activity; these data are outside the scope of the present study and will be reported separately.

Several directions follow from these observations. Mammalian models will be useful to assess efficacy in disease‑relevant contexts and to characterize exposure–response relationships. Mechanistic studies can employ EPR/spin trapping and selective scavenger competition to aid species‑level attribution of reactive equivalents [5,8,10,20–22]. Functional assays that quantify ROS dynamics, thiol oxidation state, Nrf2 pathway activity, and mitochondrial redox potential can help identify biological targets [52,53,57]. It will also be important to distinguish redox‑driven bioactivity from nonspecific effects of alkalinity or osmolality, which can be addressed with matched chemical controls and buffering strategies during assay design [58–60].

## Conclusion

In conclusion, we outline a practical framework that combines system‑level redox metrics with whole‑organism phenotyping to evaluate storable, redox‑active solutions. By bridging environmental chemistry and biomedicine, this approach supports hypothesis generation in oxidative‑stress–associated conditions and provides a foundation for subsequent mechanistic and translational studies.

## Supporting information

S1 File Raw data used for generating figures. (XLSX)
